# Secretome-Wide Analysis of Lysine Acetylation in *Fusarium oxysporum* f. sp. *lycopersici* Provides Novel Insights Into Infection-Related Proteins

**DOI:** 10.3389/fmicb.2020.559440

**Published:** 2020-09-08

**Authors:** Jingtao Li, Mingming Gao, Dean W. Gabriel, Wenxing Liang, Limin Song

**Affiliations:** ^1^Key Lab of Integrated Crop Pest Management of Shandong Province, College of Plant Health and Medicine, Qingdao Agricultural University, Qingdao, China; ^2^Department of Plant Pathology, University of Florida, Gainesville, FL, United States; ^3^Shandong Province Key Laboratory of Applied Mycology, Qingdao Agricultural University, Qingdao, China

**Keywords:** *Fusarium oxysporum*, effectors, lysine acetylation, enzymes, metabolism

## Abstract

*Fusarium oxysporum* f. sp. *lycopersici* (Fol) is the causal agent of Fusarium wilt disease in tomato. Proteins secreted by this pathogen during initial host colonization largely determine the outcome of pathogen-host interactions. Lysine acetylation (Kac) plays a vital role in the functions of many proteins, but little is known about Kac in Fol secreted proteins. In this study, we analyzed lysine acetylation of the entire Fol secretome. Using high affinity enrichment of Kac peptides and LC-MS/MS analysis, 50 potentially secreted Fol proteins were identified and acetylation sites determined. Bioinformatics analysis revealed 32 proteins with canonical N-terminal signal peptide leaders, and most of them were predicted to be enzymes involved in a variety of biological processes and metabolic pathways. Remarkably, all 32 predicted secreted proteins were novel and encoded on the core chromosomes rather than on the previously identified LS pathogenicity chromosomes. Homolog scanning of the secreted proteins among 40 different species revealed 4 proteins that were species specific, 3 proteins that were class-specific in the Ascomycota phylum, and 25 proteins that were more widely conserved genes. These secreted proteins provide a starting resource for investigating putative novel pathogenic genes, with 26 up-regulated genes encoding Kac proteins that may play an important role during initial symptomless infection stages.

## Introduction

Plants are challenged by a wide variety of microbes and parasites, such as bacteria, fungi, oomycetes, and nematodes. Thanks to their multilayered physical barriers, preformed defenses and innate immune system, plants are able to defeat most potential microbial invaders (Zhang et al., 2017; Cao et al., 2018). However, many pathogens express small, secreted proteins which are among a repertoire of pathogenicity effectors that suppress plant innate immunity (Gawehns et al., 2015). Effector functions range from altering plant cellular metabolic pathways and signaling cascades, RNA silencing, anti-microbial inhibition, and interfering with recognition machinery (Sharpee and Dean, 2016). The importance of understanding effector function has given rise to the field of “effectoromics,” which provide insights into the mechanisms underlying susceptibility to pathogens.

*Fusarium oxysporum* (Fo) is a root-infecting fungal pathogen causing wilt disease of a wide variety of plants (Michielse and Rep, 2009). The genomes of Fo are typically divided into a set of 11 “core” chromosomes containing conserved genes essential for normal development in all Fusarium species, and one or several transposon-rich and gene-poor “lineage specific (LS)” pathogenicity chromosomes (Ma et al., 2010; Gawehns et al., 2015; Vlaardingerbroek et al., 2016; van der Does et al., 2016). *F. oxysporum* f. sp. *lycopersici* (Fol), the causal agent of Fusarium wilt limited to tomato, invades the roots and subsequently colonizes the xylem vessels, thereby compromising water transport resulting in wilting of the plant (Michielse and Rep, 2009). During colonization of the tomato xylem vessels, Fol secretes small effectors, including 14 different “Secreted-in-Xylem” (SIX) proteins, which play significant roles in determining host specificity (Gawehns et al., 2015; Houterman et al., 2007; Lievens et al., 2009; Ma et al., 2013). Analysis of the Fol4287 genome reveal that most SIX effectors are species-specific and localized on LS chromosome 14, which can be horizontally transferred to a non-pathogenic strain to provide the recipient strain pathogenic capability on tomato (Ma et al., 2010; Schmidt et al., 2013). Not all effectors or predicted secreted proteins are essential for species-specific fungus-plant interactions, and only a small number of the predicted *F. graminearum* secreted proteins can be considered to be species specific effectors (Brown et al., 2012). Currently, systematic identification of potential secreted proteins beyond the LS chromosomes are poorly understood in Fol, especially during the initial infection stages before successful colonization in tomato.

The secreted proteins in principle can be covalently modified to affect their host effector functions. One frequently observed modification of proteins generally is lysine acetylation, which is a dynamic and reversible post-translational modification (PTM) occurring widely in histone and non-histone proteins (Jensen, 2006; Macek et al., 2009; Zhao et al., 2010; Finkemeier et al., 2011; Nie et al., 2015; Narita et al., 2019). Acetylation of non-histone proteins affects protein functions through diverse mechanisms, including by regulating enzymatic activity, interactions with DNA, protein stability, protein localization and crosstalk with other proteins (Narita et al., 2019). Due to the important role of lysine acetylation, the acetylomes of many eukaryotes and prokaryotes have been determined using advanced mass spectrometry (MS) and high affinity purification of acetylated peptides (Jensen, 2006; Macek et al., 2009; Choudhary and Mann, 2010; Zhao et al., 2010; Finkemeier et al., 2011; Henriksen et al., 2012; Lundby et al., 2012; Nie et al., 2015; Li et al., 2016; Lv et al., 2016; Zhou et al., 2016). However, acetylomes are typically poorly studied in plant pathogens. Until now, proteomic-based acetylomes study has only been reported in two filamentous fungi, *Botrytis cinerea* (Lv et al., 2016), *F. graminearum* (Zhou et al., 2016), and one Oomycete, *Phytophthora sojae* (Li et al., 2016). Large datasets of lysine acetylation sites were generated, which demonstrate the diverse cellular functions in these species. In addition, acetylated proteins involved in virulence were identified in *B. cinerea*, *F. graminearum*, and *P*. *sojae*, suggesting that lysine acetylation plays regulatory roles in pathogenesis (Li et al., 2016; Lv et al., 2016; Zhou et al., 2016). These studies greatly increased the knowledge of lysine acetylated proteins and expanded the global view of their functional roles in the plant pathogen landscape.

Thus far, most effectors identified from Fol were originally identified in the xylem sap of Fol- infected tomato plants. In view of these findings and the recent acetylome studies, we decided to explore in detail the secretome-wide analysis of lysine acetylation in Fol during early infection stage by mass spectrometry and various bioinformatic analysis. Gene expression levels of identified proteins were finally determined by qRT-PCR. This secretome-wide analysis of lysine acetylation would potentially give the first clues to these Kac proteins involved in the establishment/maintenance of further infection.

## Materials and Methods

### Fungal Strains, Plants, and Cultural Conditions

*Fusarium oxysporum f*. sp. *lycopersici* strain Fol4287 (Di Pietro and Roncero, 1996; Ma et al., 2010, 2013) was cultured on potato dextrose agar (PDA) plates at 25°C. To harvest the conidia, 8–10 mycelial plugs were inoculated in 150 ml of potato dextrose broth (PDB) liquid medium with shaking at 200 rpm at 25°C overnight. Tomato (*Solanum lycopersicum* cv. Alisa Craig [AC]) plants were grown in tissue culture bottle with 1/2 MS medium, which was cultured for 1 month in a climatized greenhouse at 28°C, 65% relative humidity, and a 16 h photoperiod. The harvested microconidia were suspended in 500 ml liquid minimal YEPD (0.03% yeast extract, 0.1% peptone, 0.2% dextrose) medium and 1 mg tomato roots were added to induce conidia germination. Cultures at the optimum concentration of 5 × 10^6^ conidia ml^–1^ (Vitale et al., 2019) were grown at 25°C, with shaking at 200 rpm overnight for protein or RNA extraction.

### Protein Extraction and SDS-PAGE Analysis

To harvest secreted proteins for LC-MS/MS, the overnight cultures were filtered through a double layer of miracloth and further pelleted by centrifugation at 5000 rpm for 5 min. A total of 500 ml clear culture filtrate was obtained each time, and independent biological experiments were repeated four times. The culture filtrates were concentrated to about 100 ml in a vacuum freeze dryer (ALPHA 1-2 LD plus, Martin Christ, Osterode, Germany) at −50°C, and then precipitated by adding 4 volumes of acetone, and left at −20°C overnight. The precipitates were then centrifuged at 12,000× g for 15 min at 4°C, the supernatants were discarded, and the pellets washed three times with 800 μl of cold (100%) acetone and finally centrifuged at 12,000× g for 15 min at 4°C. Pellets were desiccated using a vacuum dryer and stored at −80°C.

For SDS-PAGE (sodium dodecyl sulfate polyacrylamide gel electrophoresis), the secretome protein was dissolved in 1 × Gibco PBS (phosphate-buffered saline) buffer (Thermo Fisher Scientific), and then boiled for 5 min before loading onto 12% acrylamide gels and stained with silver. Extracted protein samples with the highest quality based on SDS-PAGE staining were then used for LC-MS/MS analysis.

### Protein Trypsin Digestion

Protein pellets from four independent biological experiments were combined and dissolved in urea buffer (8 M urea, 100 mM triethylammonium bicarbonate, pH 8.0) for trypsin digestion as described previously (Lv et al., 2016). Dissolved protein concentrations were determined with 2-D Quant kit (GE Healthcare) according to the manufacturer’s instructions. Prior to digestion, the protein solution was treated with 10 mM DTT for 1 h at 37°C and alkylated with 20 mM iodoacetamide for 45 min at 24°C in darkness. Samples were diluted by adding 100 mM (NH_4_)_2_CO_3_ to lower the urea concentration to less than 2 M. Then trypsin was added at 1:50 trypsin-to-protein mass ratio and incubated overnight for the first digestion and subsequently a 1:100 trypsin-to-protein mass ratio was incubated for a further 4 h-digestion.

### HPLC Fractionation

The trypsin digests were fractionated by HPLC (High Performance Liquid Chromatography) using Agilent 300 Extend C18 column (5 μm particles, 4.6 mm ID, 250 mm length) (Li et al., 2016). In brief, the trypsin digested peptides were separated with a gradient of 2–60% acetonitrile in 10 mM ammonium bicarbonate (pH 10.0) over 80 min into 80 fractions. Then, the peptides were finally combined into 6 fractions and further dried by vacuum centrifuging.

### Affinity Enrichment of Lysine Acetylated Peptides

For enrichment of lysine acetylated (Kac) peptides, the fractionated peptides were dissolved in NETN buffer (100 mM NaCl, 1 mM EDTA, 50 mM Tris-HCl, 0.5% NP-40, pH 8.0) and incubated with pre-washed agarose-conjugated anti-acetyl-lysine antibody beads (Cat. No. 104, PTM Biolabs, Hangzhou, China) overnight with gentle shaking at 4°C. The beads were pre-washed four times with NETN buffer and twice with ddH_2_O, respectively. Trifluoroacetic acid (0.1%) was used to elute the bound peptides from the beads. After further vacuum-dried, the obtained peptides were cleaned with C18 ZipTips (Millipore, Billerica, MA) according to the manufacturer’s instructions, followed by LC-MS/MS analysis as described previously (Zhou et al., 2016).

### LC-MS/MS Analysis

The Kac enriched peptides were dissolved in 0.1% formic acid and separated by a reversed-phase analytical column (Acclaim PepMap RSLC, 50 μm × 15 cm, 2 μm, 100 Å, Thermo Fisher Scientific). The gradient was comprised of an increase from 7 to 22% solvent B (0.1% formic acid in 98% acetonitrile) for 16 min, 22–35% for 8 min, and climbing to 80% in 2 min then holding at 80% for the last 5 min, all at a constant flow rate of 300 nl/min on an EASY-nLC 1000 UPLC (Ultra Performance Liquid Chromatography) system as described previously (Lv et al., 2016; Zhou et al., 2016).

The resulting peptides were analyzed by Q Exactive^TM^ Plus hybrid quadrupole-Orbitrap mass spectrometer (Thermo Fisher Scientific), then subjected to NSI source followed by tandem mass spectrometry (MS/MS) coupled online to the UPLC (LC-MS/MS). For MS scans, the range of m/z scan was 350–1800, and ion charge was set from +2 to +5. Fixed first mass was 100 m/z. The intact peptide was detected at a resolution of 70,000 (m/z 200). Peptides were selected for MS/MS using NCE setting as 33. Ion fragments with a resolution of 17,500 (m/z 200) were detected. A data-dependent procedure that alternated between one MS scanning followed by 16 MS scanning was applied for the top 16 precursor ions exceeding a threshold ion number of 1.5E^4^ in the MS measurement scanning with 10.0 s dynamic exclusion. The electrospray voltage of 2.0 kV was applied. Automatic gain control was used to prevent overfilling of the ion trap; and 5E^4^ ions were accumulated for generation of MS/MS spectra. The mass spectrometry proteomics data have been deposited to the ProteomeXchange Consortium via the PRIDE [1] partner repository with the dataset identifier PXD020479.

### Database Searching

The tandem MS data was processed using MaxQuant with integrated Andromeda search engine (v.1.4.2). The MS data was searched against UniProt Fol4287 (17735 sequences) database concatenated with reverse decoy database. Trypsin/P was specified as cleavage enzyme allowing up to 4 missing cleavages. The search range of mass error was set to 10 ppm for precursor ions and 0.02 Da for fragment ions. Carbamidomethylation on Cys was set as fixed modification, and oxidation on Met, acetylation on Lys were set as variable modifications. False discovery rate thresholds for protein, peptide and modification site were adjusted to 1%. Minimum peptide length was set at 7. The site localization probability was set as 0.75. All other parameters were set as default values.

### Bioinformatics

The lysine acetylated peptides and proteins were identified according to the MS/MS reports. WebLogo (Crooks et al., 2004) was employed to analyze the model of sequences constituted with amino acids in specific positions of acetyl-21-mers (10 amino acids upstream and downstream of the kac site) in all identified protein sequences. Prediction of the signal peptide was done using SignalP, and TargetP functions on the online CFGP (Comparative Fungal Genomics Platform) Server^1^. Subcellular location of protein was predicted by the PSortII analysis on CFGP (Choi et al., 2012).

Gene Ontology (GO) annotation were performed on the online server QuickGo^2^, and the proteins were classified by GO annotation based on the categories: biological process, cellular component and molecular function. The annotation protein pathway was performed according to Kyoto Encyclopedia of Genes and Genomes (KEGG). BLAST-based KO (KEGG Orthology) annotation and KEGG mapping were used to analyze the protein function pathway on the online server^3^. Gene location in chromosome of Fol4287 were performing by searching this gene sequences on NCBI server^4^. BLAST-based taxonomical distribution of homologus genes were analyzed by BLASTMatrix tool on CFGP server as well.

### RNA Extraction and qRT-PCR Analysis

To harvest Fol4287 material for RNA extraction and quantification for real-time PCR (qRT-PCR) analysis, microconidia were suspended in 100 ml liquid minimal YEPD medium and cultured with the same conditions as above. Then, Fol4287 cultures at different time points (0, 3, 6, 9, 12, and 24 h) were harvested by pelleting the developed conidia in the centrifuge at 10,000 rpm for 5 min, and finally ground in liquid nitrogen.

Total RNA was extracted from the Fol samples at different development stages using Trizol reagent (Invitrogen). Total RNA (2 μg) was used for reverse transcription with PrimeScriptTM RT reagent Kit with gDNA Eraser (Perfect Real Time) (TaKaRa). The cDNA was diluted 20-fold, and quantitative expression assays were performed by using the 2x M5 HiPer SYBR Premix EsTag (with Tli RNaseH) Reagent kit (Cat. MF787-01) with LightCycle^®^96 (Roche) real-time PCR detection system according to the manufacturer’s protocol. The relative quantification of gene expression was analyzed using 2^–ΔΔct^ method (Li et al., 2018a; Xu et al., 2018). Data were normalized against the Histone H4 gene (FOXG_09042). qRT-PCR experiment was replicated three times. The primer pairs used for real-time PCR are listed in Supplementary Data 1. Hierarchical clustering was performed using the MeV program (Li et al., 2014, 2018b). All graphs were exported by the GraphPad Prism 6 software (La Jolla, CA, United States).

## Results

### Identification and Analysis of Secretome-Wide Lysine Acetylation in Fol4287

**FIGURE 1:**
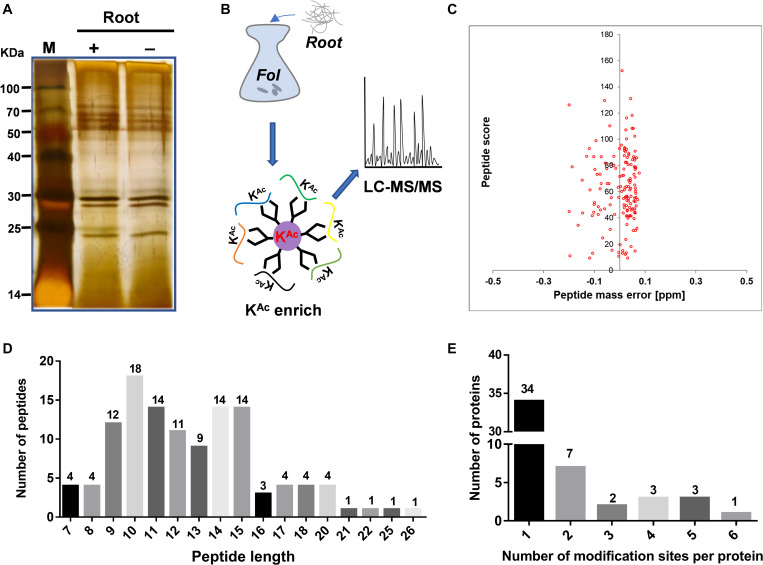
Secretome-wide identification of lysine acetylation (Kac) in *Fusarium oxysporum* f. sp. *Lycopersici* (Fol4287). **(A)** Protein extraction and SDS-PAGE analysis of secreted proteins induced in liquid YEPD with tomato root. **(B)** Schematic diagram illustrating the process of identifying the Kac proteins from the secretome by combination of affinity enrichment and LC-MS/MS. **(C)** Mass error distribution of all identified peptides. **(D)** Length distribution of the identified peptides. **(E)** Distribution of acetylated proteins based on their number of acetylation peptides. (LC/MS, mass spectrometry; SDS-PAGE, sodium dodecyl sulfate polyacrylamide gel electrophoresis; M, protein marker).

The secretome of sequenced strain Fol4287 (Ma et al., 2010) was analyzed from combined high quality protein extracts of samples of four biological replicates, each enriched for secreted protein and examined for quality on SDS-PAGE gels (Figure 1A). Then a combination of immune-affinity purification and LC-MS/MS was used to determine the predicted acetylated enriched secretome (Figure 1B). The distribution of mass errors was close to zero (Figure 1C and Supplementary Data 2) which meant the mass accuracy met the requirement for further analysis. With manually filtering insufficient peptides without Lys site or acetylation modification, the length of the most abundant peptide ranged between 9 and 15 amino acids, as expected of tryptic peptides (Figure 1D). A total of 119 lysine acetylated peptides were identified from 50 different proteins (Supplementary Data 2, 3). The distribution of acetylation sites per protein was then calculated. The acetylated proteins contained different numbers of sites ranging from 1 to 6 (Figure 1E). Among them, 68% (34) of the acetylated proteins contained only one acetylation site. The percentage of proteins with two, three, four, five, and six modification sites were 14% (7), 4% (2), 6% (3), 6% (3), and 2% (1), respectively (Figure 1E).

### Characterization of Acetylated Lysine Motifs in Fol4287

**FIGURE 2:**
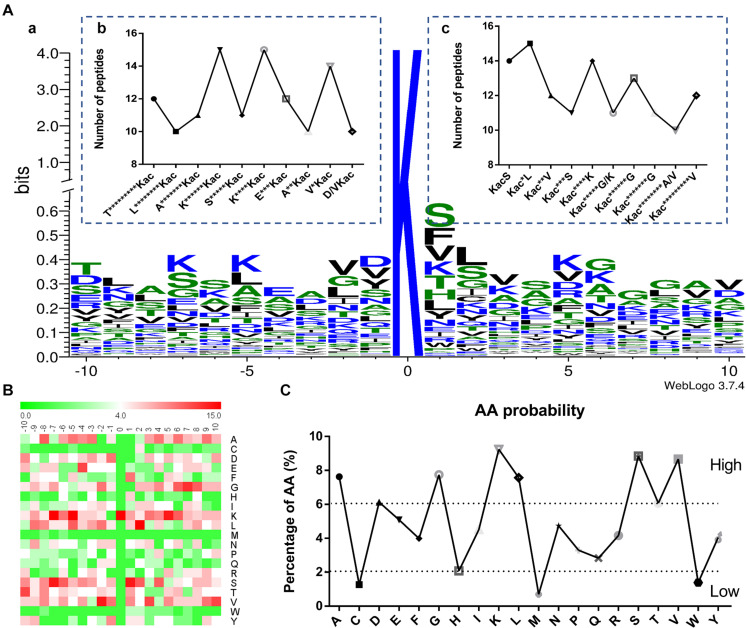
Properties of the acetylated peptides. **(A) (a)** Conservation of acetylation motif for ± 10 amino acids around the lysine acetylation sites. The size of each letter corresponds to the frequency of the amino acid residue in that position. The central K refers to the acetylated lysine. **(b,c)** Number of modification peptides in different types of acetylated peptides. **(B)** Heat map of the amino acid compositions of the acetylation sites showing the frequency of different amino acids around the modified lysine. Red indicates enrichment and green means depletion. **(C)** The amino acid properties of the acetylation sites showing the high and low frequency of amino acids around the acetylated lysine (±10).

The context of amino acids surrounding the acetylated lysine (Kac) was assessed. A total of 87 amino acid sequences of different peptides from −10 to +10 positions surrounding the Kac site were analyzed by using WebLogo program (Figure 2A and Supplementary Data 4). These sequences revealed a total of six definitively enriched motifs, namely K^******^Kac, K^****^Kac, V^∗^Kac, KacS, Kac^∗^L, and Kac^****^K (Kac indicates the acetylated lysine and ^∗^ indicates a random amino acid residue). Motifs K^******^Kac, K^****^Kac, and Kac^∗^L were the most conserved as peptides accounted for approximately 17% of all the identified peptides comparing with the random value 5%. Moreover, the heat map of amino acid residues surrounding the acetylation sites showed that the frequencies of Alanine (A), Glycine (G), lysine (K), Leucine (L), Methionine (S), and Valine (V) residues were high, whereas the frequencies of Cysteine (C), Methionine (M), and Valine (W) residues were the lowest (Figures 2B,C). These data suggested that proteins with such motifs might be preferred substrates of lysine acetyltransferases in Fol4287 cells.

### SignalP and TargetP Analyses to Predict Secretion From Fol4287 and PSORT to Predict Potential Host Cell Targeting of the Acetylated Proteins

**FIGURE 3:**
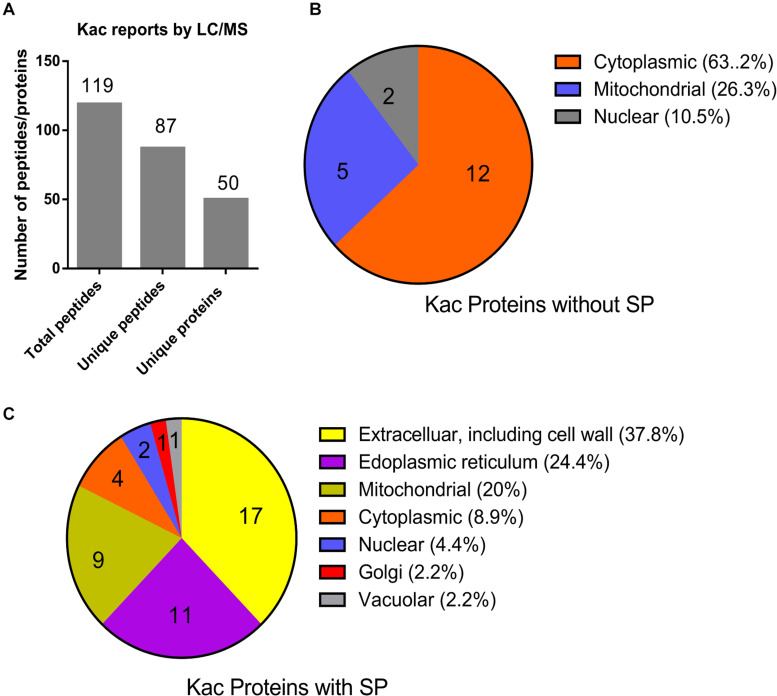
Identification and subcellular localization of the identified acetylated proteins. **(A)** Identification of the acetylated unique proteins based on the LC/MS data. **(B)** Subcellular localization of the 18 Kac proteins without SP was predicted by PSortII on CFGP platform. **(C)** Subcellular localization of the 32 Kac proteins with SP was predicted by PSortII on CFGP platform. (CFGP, Comparative Fungal Genomics Platform; SP, Signal Peptide).

From the MS data, 87 of the identified 119 peptides were unique, and matched to 50 hypothetically secreted proteins (Figure 3A and Supplementary Data 3), which accounted for 0.28% (50/17735) of the total proteins in Fol4287 (Ma et al., 2013). To confirm the likelihood of secretion of these proteins, SignalP and TargetP were used to identify 32 proteins (64%) predicted to contain the N-terminal signal peptide (SP). Eighteen proteins (36%) were predicted to be without a signal peptide. Furthermore, the predicted cellular location of these proteins was analyzed by PSORT analysis (Figures 3B,C). Those proteins without a SP were predicted to be cytoplasmic (63.2%), mitochondrial (26.3%), or nuclear (10.5%) (Figure 3B). However, the SP containing proteins were predicted to be more widely distributed, including extracellular and cell wall (37.8%), endoplasmic reticulum (24.4%), mitochondrial (20%), cytoplasmic (8.9%), nuclear (4.4%), golgi (2.2%), and vacuolar (2.2%) (Figure 3C). These findings indicate that the secreted 32 proteins with a SP have diverse biological functions prior to the initial infection of Fol.

### Functional Annotation of the 32 Fol4287 Secretory Proteins

Among the 32 proteins listed in Table 1 as potential pathogenicity effectors, eight proteins were hypothetical and uncharacterized, while 24 proteins were functionally annotated, and 83% (20/24) of these were predicted to be enzymes. The remaining four proteins were annotated as having a LysM domain (protein/carbohydrate interactions), a PAN/Apple domain (protein/protein or protein/carbohydrate interactions), Cerato-platanin (a phytotoxin), and Ferritin-Like (iron binding). Twelve proteins have multiple Kac sites as shown in Table 1.

**TABLE 1:** The secreted lysine acetylated proteins identified by LC/MS and SignalP prediction.

**Gene locus**	**Protein description**	**Protein accession**	**Kac position**	**SignalP**
FOXG_00754	Glycosyl hydrolase	A0A0D2XA39	140;249	Y
FOXG_01059	Uncharacterized protein	A0A0D2XAZ3	63	Y
FOXG_05950	Concanavalin A-like lectin/glucanase	A0A0D2XPR2	78	Y
FOXG_06166	Endonuclease/exonuclease/phosphatase	A0A0D2XQC4	116	Y
FOXG_05795	Uncharacterized protein	A0A0D2XPA7	201;218	Y
FOXG_06218	Uncharacterized protein	A0A0D2XQH2	207;362;506	Y
FOXG_08688	Uncharacterized protein	A0A0D2XXH0	196	Y
FOXG_08810	Uncharacterized protein	A0A0D2XXU1	268	Y
FOXG_03994	Aspartic proteases	A0A0D2XJ85	168;170	Y
FOXG_04049	FAD/FMN-containing dehydrogenase	A0A0D2XJD9	425	Y
FOXG_04115	N4-(Beta-N-acetylglucosaminyl)-L-asparaginase	A0A0D2XJK5	110;299	Y
FOXG_01527	Alkaline phosphatase	A0A0D2XCA4	265	Y
FOXG_02417	Peptide hydrolase	A0A0D2XET0	33	Y
FOXG_15373	LysM domain	A0A0D2YGD4	141	Y
FOXG_05199	Uncharacterized protein	A0A0D2XMM2	138	Y
FOXG_10672	PAN/Apple domain	A0A0D2Y321	132;158	Y
FOXG_04988	Inorganic pyrophosphatase	A0A0D2XM14	27;106;252;282;293;299	Y
FOXG_09102	Peptide hydrolase	A0A0D2XYN0	239	Y
FOXG_11456	Uncharacterized protein	A0A0D2Y5A1	167	Y
FOXG_11632	Amidase signature (AS) superfamily	A0A0D2Y5S6	572	Y
FOXG_11745	Phospholipase A2	A0A0D2Y637	162	Y
FOXG_13658	Histidine phosphatase	A0A0D2YBI0	75;86;100;102;245	Y
FOXG_13743	Cerato-platanin	A0A0D2YBR5	205	Y
FOXG_13854	Ferritin-like	A0A0D2YC23	103	Y
FOXG_13226	Phospholipase C	A0A0D2YAA4	343;425	Y
FOXG_13474	Carboxylesterase, type B	A0A0D2YAZ6	196;407;489;516;	Y
FOXG_13566	Glucoamylase	A0A0D2YB88	86;97;228;232	Y
FOXG_14507	Uncharacterized protein	A0A0D2YDX1	38	Y
FOXG_12372	Ribonuclease T2 family protein	A0A0D2Y7V6	110;175;181;189;194;249	Y
FOXG_16906	Gluconolactonase	A0A0C4DI40	133	Y
FOXG_16920	Alpha-amylase	A0A0D2YK52	67	Y
FOXG_16943	Glycosyl hydrolase family 17	A0A0C4DI50	214	Y

**FIGURE 4:**
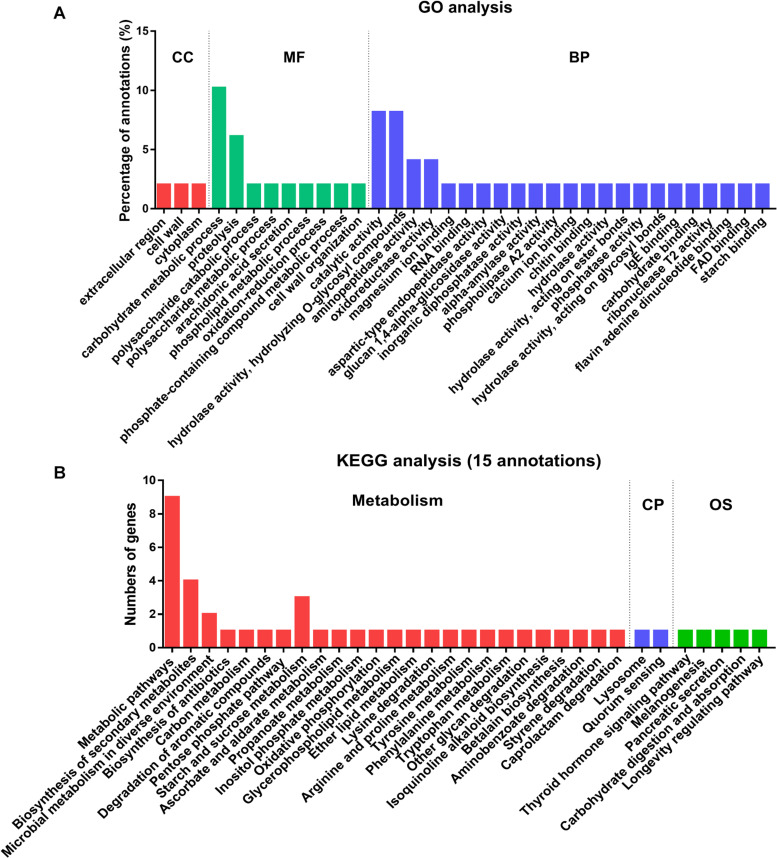
Functional classification and enrichment analysis of 32 acetylated proteins in the secretome of Fol4287. **(A)** The acetylated proteins were classified by GO annotation based on their biological process (BP), cellular components (CC) and molecular functions (MF). **(B)** KEGG pathway-based enrichment analysis of the Kac proteins. Fifteen proteins of the acetylated proteins were annotated by KEGG database and were classified by KEGG pathway.

To identify the functional processes of the 32 secreted proteins, we determined their Gene Ontology (GO) functional classifications (Figure 4A and Supplementary Data 5). Overall, these genes were mainly enzyme associated and with binding and catalytic activity categories (Figure 4A). In biological process (BP) category, most of the lysine-acetylated proteins were involved in the hydrolase and catalytic activity. In molecular function (MF) category, carbohydrate metabolism accounted for 10% of all the secreted proteins (Figure 4A). In the smallest cellular component (CC) category, only three proteins were involved, including extracellular, cell wall, and cytoplasmic regions, respectively. These results supported the earlier conclusion that most of these secretory proteins were enzymes.

A total of 15 of the secretory proteins involved in 32 KEGG pathways were found, and 85% KEGG pathways were involved in metabolism (Figure 4B and Supplementary Data 6). Among the metabolism category, 19 proteins were classified to global and overview maps cluster, 7 proteins were involved in the carbohydrate metabolism cluster, 5 proteins were involved in amino acid metabolism cluster, and 3 proteins belong to xenobiotics biodegradation and metabolites. The other proteins were in lipid metabolism, energy metabolism, glycan biosynthesis and metabolism, or biosynthesis of other secondary metabolites clusters (Supplementary Data 6). After metabolism, 5 proteins were probably involved in organismal systems (OS) and 2 proteins were involved in cellular processes (CP) pathway (Figure 4B and Supplementary Data 6). The results indicated that proteins associated with metabolism were most likely acetylated in secretome of Fol.

### Location of 32 Predicted Secreted Protein Coding Genes on the Core Chromosomes of Fol4287

**FIGURE 5:**
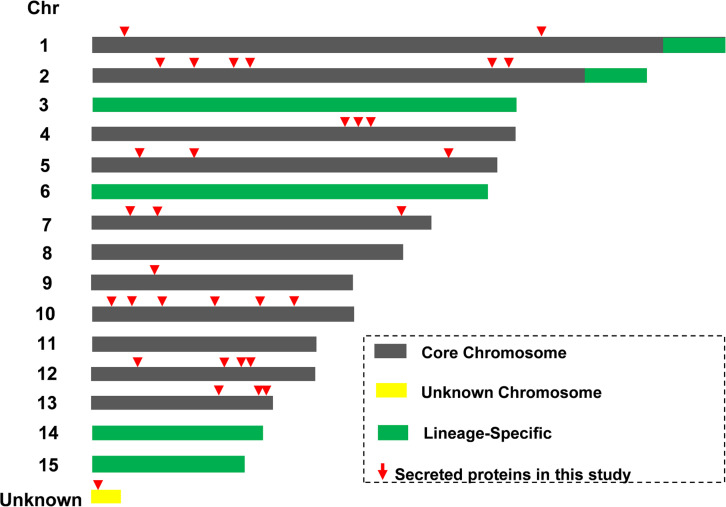
Identification of novel secreted proteins from core chromosome segments. Schematic representation of core (gray) and lineage-specific (LS, green) parts of the genome of Fol4287. Locations of 32 Kac proteins were indicated in red triangle.

The LS chromosome plays a vital role for Fol pathogenicity and all known SIX effectors are located on LS chromosome (Chr.) 14 (Ma et al., 2010; Vlaardingerbroek et al., 2016). The locations of genes encoding the Kac secreted proteins were determined (Figure 5 and Supplementary Data 7). All of the 32 secreted protein coding loci of Fol4287 were found on core chromosome genomic regions, rather than on the LS chromosome regions (Chr. 3, 6, 14, 15, end of Chr. 1, 2). No SIX proteins were found in this study. One protein (FOXG_16943) coding locus was found on a small unknown chromosome in Fol4287 based on the NCBI annotation. No predicted effectors or secreted virulence-related proteins have previously been identified on the core chromosomes.

### Homolog Scanning and Conservation Analysis of 32 Secreted Proteins in Fungi

**FIGURE 6:**
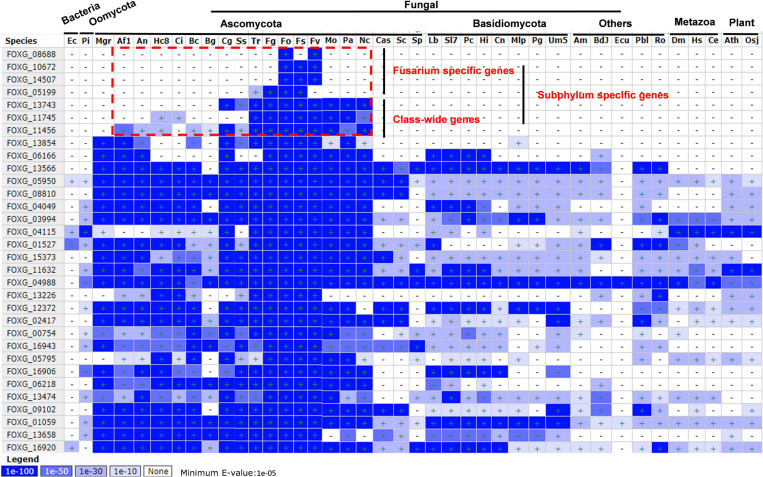
Homolog scanning and conservation analysis of 32 secreted Fol4287 lysine acetylation proteins among 40 different organisms. BLASTMatrix on CFGP was used to evaluate their conservation with *E*-value.

To reveal whether the secreted proteins were either species specific or common to a broad array of fungi, oomycetes, bacteria, metazoan, and plants, the homologs and conservation of these secreted proteins was analyzed by BLASTMatrix on the CFGP sever (Figure 6 and Supplementary Data 8). All of them have homologs in different species, however, three of them (FOXG_08688, FOXG_140672, and FOXG_14507) were Fusarium specific with high identity (*E*-value < 1E-100). The FOXG_05199 may also be considered as Fusarium specific since only one homolog was detected in *Trichoderma reesei* (Tr), but with poor identity (*E*-value = 1E-27). Three of them (FOXG_13743, FOXG_11745, FOXG_11456) have homologs in different classes, which indicated they might be class-wide in the Pezizomycotina subphylum under Ascomycota. FOXG_13854 may also be class-wide, but has conserved homologs in *Mycosphaerella graminicola* (Mgr). The remaining 24 proteins have wider distribution and conserved homologs among broad taxonomic categories (Bacteria, Oomycota, Matazoa, and Plant).

### qRT-PCR Analysis of Expression of the Kac Secreted Proteins

**FIGURE 7:**
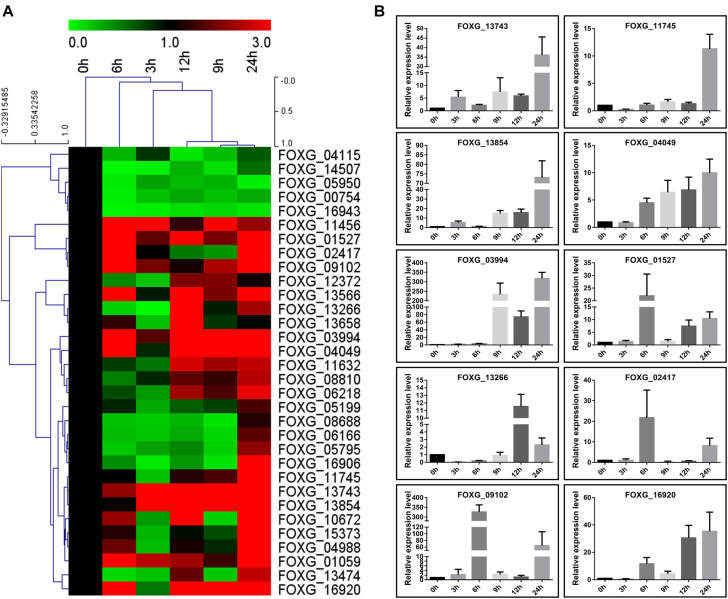
Transcription expression of 32 Kac proteins by qRT-PCR. **(A)** Hierarchical cluster of Kac proteins in transcript abundance with different development stages of conidia (0, 3, 6, 9, 12, and 24 h) induced in liquid YEPD medium with tomato root. Each gene is represented by a single row of colored boxes, and a single column represents different times during development. Induction (or repression) ranges from black to saturated red (or green) with a fold change scale bar shown up the clusters. **(B)** Quantitative RT-PCR validation of 10 high up-regulated genes (fold change > 10). *Histone 4* gene was used as internal control. The expression level means fold-changed obtained by quantitative RT-PCR (*n* = 3).

To further confirm induction of secretion of these proteins by tomato roots in liquid medium, their expression patterns were investigated in Fol4287 by quantitative reverse transcription-PCR (qRT-PCR). A total of 26 genes among the 32 genes were up-regulated (fold change > 1.5) and only 5 genes were down-regulated (fold change < 0.667) during all the conditions in response to root treatment (Figure 7A); these up-regulated genes were mainly enzymes involved in metabolic pathways in Fol (Figures 4,7 and Table 1). Among the up-regulated proteins, 12 genes were up-regulated at 6 and 9 h post infection, 16 genes were up-regulated at 12 h, and 24 genes were up-regulated at 24 h. The expression levels of only 7 genes were increased at the early stage (3 h) (Figure 7A). Thus, most of the genes positively responded to root treatment. Notably, the relative expression levels of 10 genes, *FOXG_13743* (Cerato-platanin), *FOXG_11745* (Phospholipase A2), *FOXG_13854* (Ferritin-like), *FOXG_04049* (FAD/FMN-containing dehydrogenase), *FOXG_03994* (aspartic proteases), *FOXG_01527* (Alkaline phosphatase), *FOXG_13266* (Phospholipase C), *FOXG_02417* (Peptide hydrolase), *FOXG_09102* (Peptide hydrolase), and *FOXG_16920* (Alpha-amylase), were increased more than 10 times (Figure 7B), which may crucially contribute to the initial infection of Fol.

## Discussion

Lysine acetylation (Kac) is a widespread PTM of proteins found in both eukaryotes and prokaryotes, and many virulence related proteins with Kac modification have been identified from proteomes of three fungal plant pathogens: *B. cinerea* (Lv et al., 2016), *F. graminearum* (Zhou et al., 2016), and *P. sojae* (Li et al., 2016). Given the wide variety of roles of Kac for protein function and stabilization, we hypothesized that Kac might be associated with secreted protein in Fol. Using a combination of affinity enrichment and LC-MS/MS analysis, we analyzed Kac proteins that were secreted from culture grown Fol4287 after induction using tomato roots. Overall, 32 secreted proteins with Kac modifications were identified, and 119 lysine acetylation sites were characterized. Most (68%) of the acetylated proteins contained only one acetylation site, which was similar with the previous reports in *B. cinerea* (63.5%) (Lv et al., 2016), *F. graminearum* (70.1%) (Zhou et al., 2016), and *P. sojae* (60%) (Li et al., 2016). Though Kac is a highly conserved process, little is known about the modification mechanism. There appeared to be six highly conserved Kac target motifs identified in the secretome of Fol4287, which were different from those observed in filamentous fungi *B. cinerea* (Lv et al., 2016), or *F. graminearum* (Zhou et al., 2016). Evolution and variation of different organisms may be one reason for this difference. Another possibility is the conserved Kac motifs identified in this study were from the secretome of Fol, while the motif identified from *B. cinerea* or *F. graminearum* were based on the whole proteome. However, some of the motifs found in these fungi have also been found in other organisms, indicating the conservation of Kac among different species (Mo et al., 2015; Li et al., 2016; Lv et al., 2016; Xiong et al., 2016; Zhen et al., 2016; Zhou et al., 2016; Zhu et al., 2016).

Secreted proteins/effectors play important roles for the invading pathogen through blocking plant immunity or altering specific plant processes (Zhang et al., 2017; Cao et al., 2018). In the present study, 32 proteins with an N-terminal SP were identified as induced by incubation with plant roots and most of these proteins were annotated as enzymes mainly involved in metabolism. Fusarium pathogens use both general and pathogen-specific pathogenicity mechanisms to invade their hosts (Ma et al., 2013). General pathogenicity factors are often required for proper development, mainly to acquire nutrition from various habitats, such as enzymes used for degradation of the plant cell walls composed of carbohydrates (Brown et al., 2012). Metabolism or degradation enzymes useful in diverse environments or for biosynthesis of secondary metabolites (Figure 4 and Table 1) were recovered from Fol4287, suggesting that these secretory proteins could contribute to its survival and general pathogenesis. Previous studies have also found that a large proportion of lysine acetylated proteins were categorized as metabolic proteins in other fungi and organisms (Finkemeier et al., 2011; Henriksen et al., 2012; Mo et al., 2015; Nie et al., 2015; Li et al., 2016; Lv et al., 2016; Zhou et al., 2016; Narita et al., 2019).

Specific virulence factors are employed by one or a few related Fusarium species and may include host-specific toxins and secreted effectors, such as SIX proteins from Fol4287 (Ma et al., 2013). Genome sequencing of Fol4287 identified LS chromosome regions including the ends of chromosomes 1 and 2, as well whole chromosomes 3, 6, 14, and 15, which are required for infection of tomato. Further analysis of the Fol genome revealed that 13 SIX genes were localized on chromosome 14, which is also enriched for other secreted proteins and secondary metabolite genes (Ma et al., 2010; Schmidt et al., 2013). Unexpectedly, no SIX proteins were found among the 32 secretory proteins identified in this study. It is well documented that SIX proteins are secreted during *Fol* colonizing the tomato xylem system (Ma et al., 2010) and some SIX genes are highly expressed at 72 h post inoculation (Taylor et al., 2016). In our experimental approach, proteins were identified from overnight culture under root treatment based on mass spectrometry data, which might be the main reason that no SIX protein was identified. Moreover, all 32 secretory proteins were located in the core chromosome regions rather than LS chromosome regions. Certainly, not all virulence related proteins are located in LS chromosome regions; for example, the transcription factor Sge1 was located in a core chromosome. Sge1 is required for virulence by transcriptionally connecting to pathogenicity chromosome to regulate the effector genes (van der Does et al., 2016). Besides, some effectors and effector candidates in PS (pathogen specific) regions of LS chromosomes are among the highest expressed genes in planta, and five PS transcription factors have also showed evidence of expression in planta at 72 hpi (Armitage et al., 2018). Therefore, these identified proteins from early root-inducing stage might be mainly involved in the early establishment of infection rather than colonization in planta, which need secrete specific proteins to overcoming various plant defense during infection, such as physical barriers and antifungal compounds (Michielse and Rep, 2009).

In this study, similar with some SIX proteins (Brown et al., 2012) in Fol4287 and effector molecules in *F. graminearum* (Brown et al., 2012; Ma et al., 2013), some of these identified unannotated proteins, such as FOXG_08688, FOXG_088108, FOXG_08688, FOXG_05199, FOXG_10672, FOXG_11456, FOXG_11745, FOXG_13743, FOXG_14507, FOXG_12372, were relatively small proteins (<350 amino acids), generally cysteine rich, and produced with a signal peptide for secretion. These may represent novel secreted effector candidates in Fol4287 (Supplementary Data 7). For example, a cerato-platanin protein (FOXG_13743) was found, a homolog of which was identified as involved in virulence of *Sclerotinia sclerotiorum* by targeting the plant PR1 proteins (Yang et al., 2018). This is also consistent with that only a small number of effectors are known to be essential for species-specific fungus-plant interactions of Fusarium species (Brown et al., 2012; Ma et al., 2013).

More than half of the 32 putative effectors identified in this study have homologs in other species (Brown et al., 2012). Four of these 32 proteins have homologs in other Fusarium species, and most share homologs with a broad array of fungi, oomycetes or advanced biological species. Only FOXG_08688, FOXG_10672, and FOXG_14507 have highly conserved homologs in Fusarium species like some SIX proteins (Brown et al., 2012). For example, for Avr1, Six6, Six7, Six8, and Six9, close homologs are also present in other formae speciales (Lievens et al., 2009; Chakrabarti et al., 2011; Thatcher et al., 2012), implying a more generic function for these proteins than the pathogen specific function. Among the widely distributed proteins, FOXG_15373 shared homologs in numerous other fungi, mainly due to the presence of LysM domains, a motif that is widespread in the fungal kingdom (de Jonge and Thomma, 2009; Kohler et al., 2016). For the other broadly conserved protein domains, we hypothesized that core fungal effectors may have conserved virulence functions that facilitate infections on a wide range of hosts (Kohler et al., 2016). For example, the predicted cerato-plantanin homolog FOXG_13743 might target plant PR1 to inhibit plant resistance as reported in *S. sclerotiorum* (Yang et al., 2018). The broadly conserved proteins may function as core proteins (or effectors) to serve roles in metabolism for establishment/maintenance prior to symptomless infection in the pathogen (Stergiopoulos et al., 2012; Kohler et al., 2016). Three proteins (FOXG_13266, FOXG_13474, FOXG_13566) annotated as enzymes and one small, cysteine rich protein (FOXG_14507) were located on Chromosome 12. Expression of many genes located on chromosome 12 is induced on invasion of tomato roots. Loss of chromosome 12 has limited effects on the utilization of diverse carbon sources, but resulted in reduced growth (Vlaardingerbroek et al., 2016). Taken together, only three Fusarium species specific proteins were identified and most of the novel identified proteins had widely conserved homologs, indicating that these secreted proteins were involved in general pathogenicity by conserved mechanisms. Moreover, expression of most of these widely conserved genes was up-regulated under tomato roots treatment, especially for highly up-regulated ten genes, which highlights their importance in metabolism involved in the establishment/maintenance prior to further infection.

## Conclusion

In conclusion, affinity enrichment and mass spectrometry proteomics allowed us to identify novel secreted proteins with Kac modifications. Novel secreted proteins including several effectors candidates were found beyond the LS chromosome segments and might function by general pathogenicity mechanisms.

## Data Availability Statement

The datasets presented in this study can be found in online repositories. The names of the repository/repositories and accession number(s) can be found in the article/ Supplementary Material.

## Author Contributions

JL, LS, and MG performed the experiments. JL and WL analyzed the data, wrote the manuscript, conceived the study, and provided funding. DG provided technical supports. All authors commented on the manuscript.

## Conflict of Interest

The authors declare that the research was conducted in the absence of any commercial or financial relationships that could be construed as a potential conflict of interest.

## Footnotes

1 http://cfgp.riceblast.snu.ac.kr/main.php

2 https://www.ebi.ac.uk/QuickGO/

3 https://www.kegg.jp/kegg/kegg1a.html

4 https://www.ncbi.nlm.nih.gov/gene/
